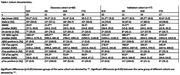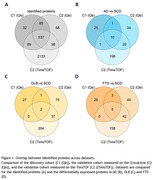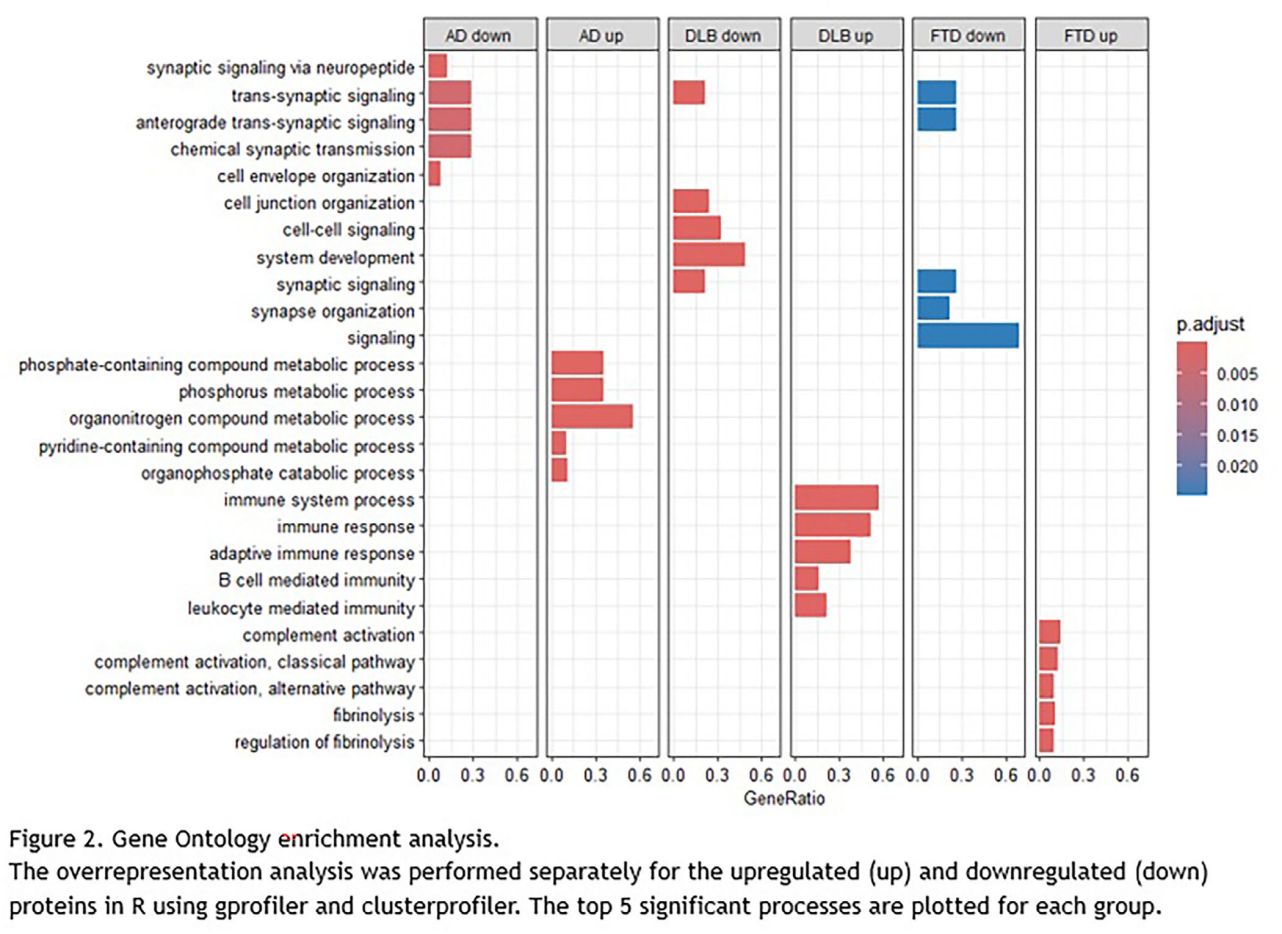# Discovery of dementia type specific biomarkers with untargeted CSF proteomics

**DOI:** 10.1002/alz.095441

**Published:** 2025-01-09

**Authors:** Marijke E. Stokkel, Lisa Vermunt, Jaco Knol, Sander Piersma, Thang V. Pham, Berend Gagestein, Richard R. de Goeij de Haas, Afina Willemina Lemstra, Yolande A.L. Pijnenburg, Pieter Jelle Visser, Betty M. Tijms, Charlotte Teunissen, Connie R. Jimenez

**Affiliations:** ^1^ Neurochemistry Laboratory, Department of Laboratory Medicine, Vrije Universiteit Amsterdam, Amsterdam UMC, Amsterdam, North Holland Netherlands; ^2^ Alzheimer Center Amsterdam, Neurology, Vrije Universiteit Amsterdam, Amsterdam UMC location VUmc,, Amsterdam, North Holland Netherlands; ^3^ Neurochemistry Laboratory, Department of Laboratory medicine, Vrije Universiteit Amsterdam, Amsterdam UMC location VUmc, Amsterdam Netherlands; ^4^ Alzheimer Center Amsterdam, Neurology, Vrije Universiteit Amsterdam, Amsterdam UMC location VUmc, Amsterdam, North Holland Netherlands; ^5^ OncoProteomics Laboratory, Department of Medical Oncology, Amsterdam UMC (VUmc), Amsterdam, North Holland Netherlands; ^6^ Alzheimer Center Amsterdam, Neurology, Vrije Universiteit Amsterdam, Amsterdam UMC location VUmc, Amsterdam Netherlands; ^7^ Alzheimer Center and Department of Neurology, Amsterdam Neuroscience Campus, VU University Medical Center, Amsterdam Netherlands

## Abstract

**Background:**

Different pathologies can cause dementia, including Alzheimer’s disease (AD), dementia with Lewy bodies (DLB) and frontotemporal dementia. Understanding the biological mechanisms underlying these diseases is important in order to develop therapies. Here we performed cerebrospinal fluid (CSF) proteomics in AD, DLB and FTD in order to study proteomic changes and identify novel potential biomarkers.

**Method:**

Individuals from the discovery (n = 80) and validation (n = 77) cohort were selected from the Amsterdam Dementia Cohort based on clinical diagnosis and availability of a CSF sample. Single shot nano‐LC‐MS/MS was performed using a data independent acquisition (DIA) method in a QExactive HF (Qe) mass spectrometer. Additionally, the validation cohort was remeasured on a newly acquired EVosep‐TimsTOF‐HT (Bruker) allowing for deeper proteomic profiling. For each cohort, protein intensities were log2 transformed, and scaled according to the mean and standard deviation of the SCD group. Next, all groups were compared to each other on the levels for each protein with linear regression models, including age and sex as covariates. We performed Gene Ontology enrichment analysis for the differentially expressed proteins in the TimsTOF dataset.

**Result:**

In total we identified 3501 unique proteins, 803 in the discovery cohort, 1296 in the Qe measurements of the validation cohort, and 2873 in the recent TimsTOF remeasurements of the validation cohort (Figure 1A). We identified 37, 30, and 34 differentially expressed proteins in AD, DLB and FTD, of which 11, 3 and 8 replicated in the Qe measurements cohort (Figure 1B‐D). Proteins that replicated between cohorts included ALDOA, SMOC1, CHI3L1 and PKM for AD, NPTX2 and VGF for DLB and SERPINA3 and CHI3L1 for FTD. Investigation of the most statistically enriched biological terms shows processes related to synaptic signaling to be downregulated in AD, DLB and FTD. Similar to literature (Bader et al., 2020), metabolic processes were found to be upregulated in AD. In contrast, upregulation of adaptive immune responses in DLB and complement activation and fibrinolysis in FTD have not been previously observed (del Campo et al., 2023; van der Ende et al., 2019)

**Conclusion:**

Improvements in mass spectrometers enable deeper proteome profiling, essential for uncovering novel processes underlying disease.